# Unveiling the Power of Deuterium in Drug Discovery: A Comprehensive Overview

**DOI:** 10.1002/mco2.70799

**Published:** 2026-06-07

**Authors:** Mukta Lele, Ajit Manchare, Swapnali Parit, Amol D. Gholap, Krishna Jadhav, Navnath Hatvate, Keshav Raj Paudel, Satish Rojekar

**Affiliations:** ^1^ Institute of Chemical Technology, Marathwada Campus Jalna Maharashtra India; ^2^ Department of Pharmaceutics St. John Institute of Pharmacy and Research Palghar Maharashtra India; ^3^ Institute For Bioengineering of Catalonia (IBEC) Barcelona Spain; ^4^ NICM Health Research Institute & School of Science Western Sydney University Westmead NSW Australia; ^5^ Woolcock Institute of Medical Research Macquarie University Sydney New South Wales Australia; ^6^ Institute for Translational Medicine and Pharmacology, Department of Pharmacological Sciences Icahn School of Medicine at Mount Sinai New York New York USA

**Keywords:** deucravacitinib, deuterated drugs, deuterium, drug metabolism, pharmacokinetic properties

## Abstract

Deuterium, the heavy isotope of hydrogen, has unfolded as a cornerstone in modern drug discovery due to its potential to influence metabolic stability and pharmacokinetic behavior. The deuterium kinetic isotope effect (KIE), which strengthens carbon–deuterium bonds, make it possible to improve therapeutic efficacy while maintaining pharmacological activity. Although some deuterated drugs, notably donafenib and deutetrabenazine, have demonstrated clinically significant efficacy, their limited use is an effect of ongoing challenges with metabolic switching and species‐specific variation, as well as inadequate mechanistic understanding. This review presents a systematic discussion of the recent innovations in site‐selective deuteration, the principles that underpin the KIE process, and the effects of deuterium substitution on drug metabolism, toxicity, and blood–brain barrier penetration. It illustrates novel implications in oncology, rare diseases, and central nervous system disorders, as well as the integration of deuterated chemistry with modalities such as proteolysis‐targeting chimaeras, peptides, and nucleic acid therapeutics. Furthermore, the review's discussion includes the current challenges, synthesis, analytical limits, and regulatory considerations that influence further development. Overall, the review offers a strategic roadmap for utilizing deuterium‐enabled molecular engineering to accelerate the development of next‐generation precision medicine, guiding rational design, innovation toward safer, longer‐lasting, and more effective treatments.

## Graphical abstract

The role of deuterium replacement in drug discovery, its progress, opportunities, and challenges.

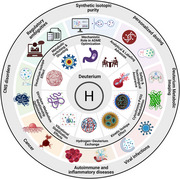

## 1 Introduction

**FIGURE 1:**
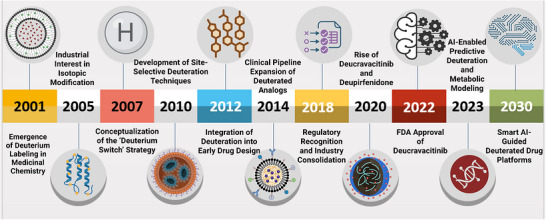
Timeline for deuterium in drug discovery. The chronological development of deuterium in drug discovery, starting from early interest in isotopic labeling and the introduction of the “deuterium switch” strategy to its integration into modern drug design and clinical development. It highlights key milestones, including regulatory recognition, US FDA approval of drugs such as deucravacitinib, and prospects involving artificial intelligence (AI)‐driven deuterated drug design and predictive metabolic modeling.

Deuterium is a stable isotope of hydrogen with one proton, neutron, and electron. It constitutes only 0.015% of the total hydrogen present in the natural environment, rendering it rare. At these naturally occurring levels, hydrogen combines with oxygen to form standard water, commonly called light water. This change may seem minor, but it has broader implications for the molecule's chemistry and its biological effects. Compared with its counterpart, that is, hydrogen–deuterium and its analogues, it has greater market potential and applications, serving as a crucial agent in pharmaceuticals [1, 2]. Nonetheless, heavy water serves as a powerful neutron moderator in nuclear reactors, and it is produced when the deuterium content in water is increased. One of these is the CANDU (Canada deuterium uranium) reactor, where the “D” stands for deuterium [3, 4]. The first deuterated drug approved by the United States Food and Drug Administration (US FDA) in 2017 is deutetrabenazine. It is a vesicular monoamine transporter (VMAT) 2 inhibitor and structurally similar to its counterpart, tetrabenazine. Drug metabolism is found to be altered, with a prolonged half‐life, without any significant change in pharmacological effect, resulting in excellent patient compliance [5, 6]. The second important milestone in the deuterium journey to the drug market was the development of the novel deucravacitinib, an allosteric tyrosine kinase 2 (TYK2) inhibitor that received approval for the treatment of psoriasis in September 2022 (Figure 1) [7].

The de novo deuterated prospects in clinical trials include VX‐984, deucrictibant, and two follow‐up medicines for deucravacitinib (BMS‐986202 and BMS‐986322). In addition to these advanced candidates, there are other instances in the literature where deuterium is employed early in the R&D process to overcome pharmacokinetic obstacles and possibly enhance other therapeutic attributes [8, 9, 10]. These include MNK1/2 protein degraders for triple‐negative breast cancer, dual TYK2/JAK1 inhibitors for autoimmune diseases, Mpro inhibitors for COVID‐19, memantine analogues for Alzheimer's disease, PET ligands for Huntington's disease, and inhibitors of the MDM2–p53 protein–protein interaction for cancer [11, 12, 13, 14]. This deuterium and its analogues can be explored further in the field of pharmaceutical care.

The stability of the deuteron is essential for the development of drugs. If neutrons could not maintain stability within deuteron structures, they would have decayed long ago [15]. Chemical bonds generated by deuterium and tritium are significantly more stable than those created by ordinary hydrogen (protium). Compared with deuterium, which has substantially higher values, normal hydrogen has a lower triple point, boiling point (point of vaporization), heat of fusion, and heat of vaporization [16, 17]. Deuterium gas lacks color but emits a noticeable pink light when ionized. Due to stronger bonding, heavy water is approximately 10.6 times denser than regular water (with a density of 1.105 g/cm^3^). Heavy water causes ordinary water ice to float, whereas normal water causes heavy water ice to sink [18, 19].

Absorption, drug distribution, metabolism, and excretion (ADME) can all be impacted by deuterium substitution. Deuterium‐containing drugs have the potential to produce longer‐lasting therapeutic effects, lower dosing frequency, and better patient compliance by slowing metabolism or altering clearance rates. Deuterium can alter a drug's pharmacodynamic characteristics, affecting how it interacts with biological processes and target receptors [6, 20, 21]. This alteration may increase the therapeutic index, reduce side effects, and enhance pharmacological efficacy. It may also enable more effective targeting of particular tissues or cell types more successfully [22].

Researchers are actively pursuing advancements and innovations in deuterium‐containing pharmaceuticals across diverse domains. A primary focus is the systematic optimization of metabolic stability by comprehensively understanding deuterium's influence on drug metabolism. The primary objective is to investigate a paradigm shift in drug design, which may lead to enhanced therapeutic efficacy. Concurrently, strides are being made in the implementation of precision medicine principles [23, 24]. The development of specific inhibitors or mimics for lncRNAs and miRNAs, alone or in combination with conventional chemotherapy, is currently emerging as a promising strategy [25].

A notable effort is the development of long‐acting formulations for deuterium‐containing drugs. Researchers are striving to engineer formulations that prolong drug action, thereby ensuring sustained therapeutic effects and, in turn, fostering improved patient adherence to prescribed regimens. Advances are being made in predicting and comprehending the safety profiles of deuterium‐containing drugs. This includes advances in toxicology testing and metabolite profiling, which contribute to a more comprehensive understanding of safety and tolerability factors [6, 26]. The unveiling of new therapeutic domains indicates a growing scope of deuterium‐containing drugs. The prospect for these drugs to transcend conventional classifications and meet unmet medical needs grows as researchers identify novel applications. Despite the fact that some of the deuterated drugs have already been launched into the market and numerous others are being examined in clinical studies, it is important to have a complete overview of their chemical background, pharmacokinetic benefits, usage in therapy, and future outlook [27]. Thus, the purpose of the review is to organize and discuss the current developments and advances in the design, synthesis, pharmacological importance, and clinical development of deuterium‐containing drugs in contemporary drug development.

This review gives a summary of the basic chemistry of deuterium and its isotopic characteristics in drug design. It talks about the history of deuterated drugs and their evolution as instruments of analysis to drugs. The review also gives a summary of the effect of deuterium substitution on pharmacokinetics, metabolic stability, and drug safety. Significant illustrations of approved and investigational deuterated drugs are presented to explain its clinical applications. Moreover, the new areas of therapy, new approaches to drug design, and perspectives of deuterium‐based drugs are pointed out. All in all, deuterium labeling of drug molecules is a novel approach to control pharmacodynamics and pharmacokinetics without drastically modulating the structure of therapeutically active drugs. Since the initial application of deuterium in analytical chemistry to the recent passing of deuterated drugs, including deutetrabenazine and deucravacitinib, deuterium has shown a lot of potential in enhancing the effectiveness, safety, and compliance of drugs. As synthetic chemistry, metabolite profiling, and precision medicine continue to develop, deuterium‐based drug design will come into the focus of more and more significant functions in addressing the drawbacks related to traditional therapeutics. Therefore, it is necessary to know the principles, application and future perspectives of deuterium incorporation in order to pursue the further development of modern drug discovery.

## 2 Deuterium in Drug Discovery: An Overview

### 2.1 Historical Perspective of Deuterated Compounds

The heavy‐weight stable isotope of hydrogen, deuterium, has emerged as a breakthrough in drug discovery due to its subtle yet significant effects on the pharmacokinetics and toxicity of bioactive compounds. Deuteration (the replacement of hydrogen with deuterium) can enhance the efficacy and safety of drugs by altering their metabolic stability and/or reducing unwanted side effects, leveraging the kinetic isotope effect (KIE) without altering the overall molecular structure [6, 28]. In the past, evidence‐based research has been conducted on the so‐called deuterium switch strategy, which involves producing analogues of existing drugs, such as deutetrabenazine, approved by the US FDA in 2017. More recently, new synthetic methodologies have enabled the de novo design of deuterated new chemical entities, such as deucravacitinib, marking a shift in paradigm toward new applications of deuterium in medicinal chemistry [5, 6, 29].

The historical perspective of deuterated compounds traces back to the mid‐20th century when researchers began exploring the unique properties of deuterium, a stable isotope of hydrogen [30]. Deuterium has a proton and a neutron in its nucleus, making it twice as heavy as regular hydrogen. One notable milestone in the early usage of deuterated chemicals is the development of deuterated solvents [31]. Deuterated solvents, such as deuterated chloroform and deuterated dimethyl sulfoxide, have become common in nuclear magnetic resonance (NMR) spectroscopy [30]. Deuterium's distinctive characteristics enabled higher resolution and signal‐to‐noise ratios in NMR experiments, enhancing the precision of chemical analysis [32].

The early adoption of deuterated compounds in pharmaceutical industry attracted interest due to their possible influence on drug metabolism and pharmacokinetics. Researchers examined deuterium substitution in numerous drug molecules to influence stability, bioavailability, and metabolic pathways. Notably, deuterium incorporation addressed issues such as rapid metabolism and undesired side effects of certain drugs [6]. One of the pioneering examples is development of drugs, including the use of deuterium in antiviral (e.g., deuterated telaprevir) and anticancer (e.g., donafenib (d3‐sorafenib)) medications [33, 34]. As research progressed, deuterated compounds found applications beyond the pharmaceutical industry. For instance, deuterium labeling became a valuable tool in tracing molecular pathways in biochemical and environmental studies [35].

In the specialized field of deuterated volatile anesthetics (e.g., deuterated sevoflurane), for instance, patents were issued from files as late as 1995, or around 18 years after Dow's first filing upon deuterated anesthetics. A variety of related and unrelated patents were later obtained for various purposes. From 1998 to 2009, Isotechnika's inventors were granted permission for many patents pertaining to medication deuteration; the firm then concentrated on voclosporin [23, 36]. Then, starting in 2005, a number of inventors, including Tung, Gant, and Czarnik, submitted a lengthy series of patent applications claiming deuterated versions of already‐marketed nondeuterated medications, with assignments to Concert, Auspex, and Protia, Deuteria, and Deuterx, in that order. These firms have lately issued patents in 2013 and 2014 that include deuteration claims [3, 23, 37]. This tactic has been well documented and has undoubtedly been a major factor in its success.

### 2.2 Deuterium and Its Isotopic Effects

Isotopic effects are an expression of the quantum nature of nuclei; compounds with different hydrogen isotope compositions exhibit distinct vibrationally averaged characteristics due to zero‐point fluctuations. H/D isotope effects on the NMR chemical shifts of nuclei participating in a hydrogen bond offer crucial information on the proton position inside the bridge. When the bridging deuteron is protected from the proton, a positive primary isotope effect is often taken to signify a strong hydrogen bond with a double‐well potential [38].

### 2.3 Deuterium Labeling and Pharmacokinetics

Deuterium labeling, a sophisticated technique used in pharmacokinetics and drug development, is employed to investigate drug behavior in the human body. This approach involves replacing hydrogen atoms with deuterium, a stable isotope of hydrogen that causes minimal alteration to the chemical properties or biological activity of the drug [39]. The introduction of deuterium at specific positions in the drug molecule allows researchers to track the ADME pathways, which is particularly useful for elucidating metabolic processes and identifying metabolites, which help to define the safety, efficacy, and possible toxicity of a drug [35, 40, 41]. Deuterium labeling is particularly useful for determining drug metabolism because it provides more sensitive and accurate information than traditional methods [40]. In addition, deuterium‐labeled drugs may have different pharmacokinetic profiles, such as longer half‐lives, which can result in prolonged drug action and lower dosing frequency. In clinical trials, deuterium‐labeled drugs are evaluated for their pharmacokinetic behavior in humans, and regulatory bodies like the US FDA may need to be more vigilant in confirming the safety and efficacy of these compounds [23, 29].

## 3 Progress in Deuterium‐Containing Drugs

The development and approval of novel therapeutics exhibiting improved pharmacokinetic and safety characteristics through strategic deuteration have been a milestone in the progress of deuterium‐containing drugs. Since the US FDA approved deutetrabenazine for use in Huntington's disease, the pipeline has seen the addition of deuterated analogues of enzalutamide, ivacaftor, donafenib, and deucravacitinib, each with certain advantages, including greater metabolic stability, reduced toxicity, and enhanced therapeutic activity. There are many candidates, such as those for cancer, neurodegenerative diseases, and COVID‐19, in the process of clinical trials, and this can be considered a paradigm shift in drug creation due to the specific incorporation of deuterium in the pharmacological field [34, 42, 43].

### 3.1 Case Studies of Deuterated Drug

The deuterated drugs are studied for different indications and are represented as case studies of approved and investigational deuterated drugs in Table 1.

**TABLE 1:** Representative case studies of deuterated drugs.

Deuterated form drug	Parent drug	Structure	Indication	Remark	References
C20‐D3‐retinyl acetate	Retinyl acetate	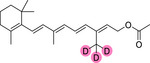	Stargardt disease	Enhanced bioavailability and efficacy in vitamin A supplementation, with reduced risk of degradation and metabolic conversion	[44]
AVP‐786 (d6‐dextromethorphan + quinidine)	Dextromethorphan/quinidine	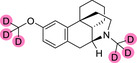	Agitation in patients with dementia of the Alzheimer's type	Better management of conditions like cough or neurological disorders with a potentially lower risk of adverse effects	[45]
Deupirfenidone (d3‐pirfenidone, SD‐560, LYT‐100)	Pirfenidone	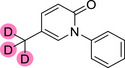	Idiopathic pulmonary fibrosis	Enhanced bioavailability and extended half‐life, leading to potentially improved efficacy and reduced dosing frequency	[46]
PXL065 (DRX‐065)	Pioglitazone	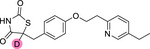	Nonalcoholic steatohepatitis	Better glycemic control with a reduced risk of side effects associated with high doses or frequent dosing	[47, 48]
Deutivacaftor (d9‐ivacaftor, CTP‐656, VX‐561)	Ivacaftor	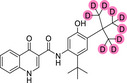	Cystic fibrosis	Better maintenance of therapeutic levels with potentially reduced dosing frequency, thereby improving patient adherence and treatment outcomes in cystic fibrosis management	[49]
Deutenzalutamide (d3‐enzalutamide, HC‐1119)	Enzalutamide	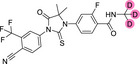	Metastatic castration‐resistant prostate cancer	Improved metabolic stability, and reduced variability in plasma concentrations, which could result in better therapeutic efficacy and a more predictable dosing regimen in the treatment of conditions like prostate cancer	[50]
RT001 (d2‐linoleic acid ethyl ester)	Linoleic acid	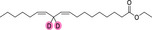	Friedreich ataxia, Skin ageing	Prolonged therapeutic effects, reduced dosing frequency, and improved tolerability, making RT001 a promising option for conditions such as dry eye syndrome or cosmetic applications	[51]
Donafenib (d3‐sorafenib)	Sorafenib	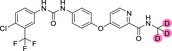	Metastatic colorectal cancer, advanced hepatocellular carcinoma	Enhanced bioavailability, metabolic stability, and tissue distribution, which could lead to better efficacy and tolerability in the treatment of conditions like hepatocellular carcinoma	[52]
VV116 (JT001)	Remdesivir	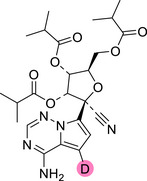	COVID‐19	Exhibit a broader spectrum of antiviral activity or a more favorable safety profile, making it a promising alternative for the treatment of viral infections such as COVID‐19	[53]
Deuruxolitinib (d8‐ruxolitinib; CTP‐543)	Ruxolitinib	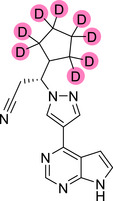	Alopecia areata, renal impairment, hepatic impairment	Enhanced bioavailability, extended half‐life, and reduced metabolic liabilities	[54]
CTP‐499 (PCS499)	Major metabolite of pentoxifylline	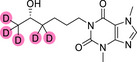	Type 2 diabetes mellitus; chronic kidney disease	Enhanced bioavailability and metabolic stability, leading to more consistent drug levels and potentially improved therapeutic efficacy in conditions such as peripheral artery disease or chronic kidney disease	[55]
Deutarserine (d‐D‐serine, CTP‐692)	d‐Serine	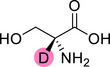	Schizophrenia (NCT04158687)	Better therapeutic efficacy, prolonged duration of action, and potentially fewer adverse effects, making deutarserine a promising option for conditions such as schizophrenia or neurological disorders	[6]
Deudomperidone (CIN‐102)	Domperidone	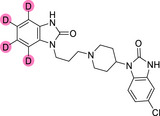	Gastroparesis (NCT05832151)	Better therapeutic efficacy, prolonged duration of action, and potentially fewer adverse effects, making deutarserine a promising option for conditions such as schizophrenia or neurological disorders	[56]
Deuterated indiplon	Indiplon	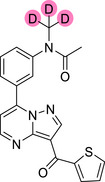	Treatment of insomnia	Improved efficacy, and potentially reduced dosing frequency, offering a promising option for the treatment of insomnia with better patient compliance and tolerability	[57]
CTP‐221 (deuterated (S)‐lenalidomide)	Lenalidomide (revlimid)	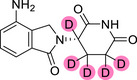	Multiple myeloma and in myelodysplastic syndromes (MDS)	Result in better treatment efficacy, prolonged duration of action, and potentially fewer adverse effects, providing a promising alternative for conditions such as multiple myeloma or myelodysplastic syndromes	[58]
Deuterated odanacatib	Odanacatib	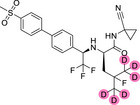	Treatment of postmenopausal osteoporosis	Improved pharmacokinetic properties such as enhanced metabolic stability, prolonged half‐life, and optimized bioavailability	[59]
Deuterated teleprevir	Teleprevir	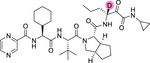	Treatment of hepatitis C infection	Improved metabolic stability compared with teleprevir, potentially leading to a longer half‐life and enhanced efficacy in treating hepatitis C infection	[60]
CTP‐518	Atazanavir	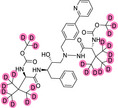	Treat infection of human immunodeficiency virus (HIV) (NCT01458769)	Enhanced bioavailability and metabolic stability, potentially leading to better treatment efficacy and reduced risk of drug‐drug interactions in HIV management	[61]
SD‐254	Venlafaxine	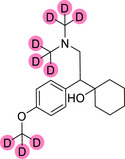	Antidepressant of the serotonin–norepinephrine reuptake inhibitor (SNRI)	Improved pharmacokinetic properties, including enhanced bioavailability and prolonged half‐life, which could result in more consistent drug levels	[1]
BDD‐10103	Tolperisone	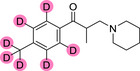	Centrally acting muscle relaxant	Enhanced efficacy or reduced side effects in the treatment of muscle spasms or related conditions, potentially resulting from its unique pharmacological profile or improved selectivity for specific molecular targets	[8, 62]

### 3.2 Benefits of Deuterium Incorporation in Drug Candidates

Currently, marketed pharmaceutical substances undergo metabolic changes mediated by cytochrome P450 (CYP) family enzymes in approximately 70–80% of drugs in clinical practice. Intermediate and final metabolites produced by this metabolic process may be unstable, reactive, nonselective, and toxic intermediate [63]. To improve the pharmacokinetic performance of drugs, developers commonly replace hydrogen (H) with deuterium (D) based on the KIE [64, 65]. However, caution must be exercised because deuteration at one location can alter the compound's biotransformation pathways, potentially increasing metabolism at other locations. This phenomenon is known as metabolic switching, where an unintended metabolic route becomes more prominent. Furthermore, a metabolic pathway that produces desired and nontoxic metabolites may be increased through a process known as metabolic shunting [6, 66, 67]. This is illustrated in preclinical examples, such as nevirapine and clinical examples, like CTP‐347. Following deuteration, the metabolic profile changes in both cases, producing different quantities of metabolites without significantly affecting the total metabolic rate [68].

The deuteration of caffeine, specifically at individual methyl groups, has been observed to diminish oxidative processes while concurrently inducing noteworthy metabolic switching at sites where deuteration is absent. Comprehensive deuteration targeting all three methyl groups, as illustrated by d9‐caffeine, can reduce N‐dealkylation reactions and improve the pharmacokinetic profile. This deuterated variant requires lower doses and reduced dosing frequency, thereby offering as a potential clinical option for caffeine [69]. For the drug approved for treating cystic fibrosis, deuteration of ivacaftor also improved the pharmacokinetic and prevented metabolic switching. Ivacaftor has two tert‐butyl moieties, and its oxidative metabolism is primarily directed toward one of them; soft deuteration of the tert‐butyl moiety was achieved (d9‐ivacaftor), and no major instances of metabolic switching were observed in the other tert‐butyl moiety. Deutivacaftor has the potential to be administered once a day rather than twice a day as is required for ivacaftor [70]. The incorporation of deuterium into a drug can result in significant changes in its metabolic profile, such as changes in the ratio of the parent drug to its metabolites and the quantities of formed metabolites. While unique metabolites have not been identified for the all‐hydrogen analog, there have been reports of decreased rates of metabolism and decreased incidences of metabolic switching that can impact the pharmacodynamics, tolerability, and efficacy of deuterated drugs [8].

### 3.3 Role of Deuterated Drugs on Efficacy and Safety

The principal effect of deuteration is a considerable reduction in systemic clearance rates, which extends the compound's biological half‐life. The drug's duration of action rises proportionately as systemic clearance decreases, this offers potential therapeutic advantages, including the capacity to maintain identical systemic exposure. This phenomenon can boost efficacy and alleviate side effects, depending upon the drug's specific pharmacodynamic/pharmacokinetic properties [68]. The administration of deuterium in drug therapy has demonstrated minimal adverse effects in mammals and has been safely administered to healthy individuals without reported complications. However, the impact of deuteration on specific drug molecules is less predictable, and the pharmacokinetic changes induced by deuteration may lead to unforeseen adverse effects [29]. Deuterated compounds may exhibit a more challenging elimination process than anticipated. Deutetrabenazine holds the distinction of being the first deuterated compound approved for clinical use, yet some earlier compounds were abandoned due to unexpected toxicities. It is important to note that not all drugs are benefited by deuteration, leading to ongoing debates regarding the role of deuterated drugs in the pharmaceutical marketplace and the extended patentability of deuterated versions compared with their nondeuterated counterparts [29]. Examples of advances in deuterium drug development include deutivacaftor, used to treat cystic fibrosis; and donafenib, used to treat hepatocellular carcinoma, which demonstrate improved metabolic stability and efficacy. Case studies have demonstrated several advantages, including improved pharmacokinetics with longer half‐lives, lower dosing rates, and reduced toxic metabolites resulting from metabolic shunts. These developments underscore the importance of deuteration in enhancing the safety, efficacy, and bioavailability of drugs in a wide variety of clinical applications [27, 34].

## 4 Safety and Toxicology

Deuterium replacement in the drugs does not have a high level of systemic toxicity, and the literature supports safe levels of exposure in animals, both in humans and in sensitive groups, such as, but not limited to, pregnant women or neonates [71, 72, 73]. The toxicological profiles are mostly favorable due to the small amount of deuterium found in pharmaceuticals. Unpredictable pharmacokinetic fluctuations may cause metabolic shunting or switching, leading to new toxicity or a change in the metabolite profile, as observed in some deuterated drug candidates. Although the side effects of using deuterated drugs are minimal, deuterated drugs, such as deutetrabenazine, are safer due to their improved metabolic stability and longer half‐lives, resulting in fewer side effects and highlighting their promising safety and therapeutic profiles in clinical use [29, 34].

An effect of deuterium on metabolites can be explained by an example like the precursor of catecholamine neurotransmitters (dopamine, norepinephrine, and epinephrine), tyrosine is an amino acid found in protein. Tyramine shares structural similarities with norepinephrine and adrenaline and exhibits comparable physiological effects, known as “pressors,” including elevated blood pressure, vasoconstriction, and increased heart rate. The liver and stomach enzyme MAO (monoamine oxidase) normally breaks down ingested tyramine. Tyramine‐containing meals can cause the development of severe hypertension when taken with MAO inhibitor medications [74]. Belleau et al. found that deuterated tyramine was metabolized more slowly in cats than nondeuterated tyramine in one of the first experimental examples of the influence of deuteration on drug metabolism. Consequently, a longer pressure response was linked to deuterated tyramine [75].

One practical use of deuterium substitution involves the inhibition of drug metabolism, particularly in the context of CYP450‐mediated transformations. Within the extensive range of reactions facilitated by this superfamily, dealkylations of ethers and amides typically exhibit high KIEs (>2). In contrast, amine N‐dealkylation is less influenced (<2), although the reasoning behind this phenomenon remains a topic of ongoing debate. In such cases, precise deuteration depends solely on a secondary KIE [6, 64, 65]. The impact of deuterium integration on enzymatic oxidizing transformations has garnered attention, extending beyond CYP450. For instance, aldehyde oxidase (AO) participates in the oxidation of the formyl carbon in aldehydes, as suggested by its name. Additionally, AO plays a role in the oxidation of α‐carbons adjacent to nitrogen in heteroaromatic ring systems (e.g., pyridines, pyrimidines, benzimidazoles), involving a rate‐limiting C−H bond cleavage. This characteristic makes AO amenable to precise deuteration, as exemplified by carbazeran and zoniporide [76, 77]. A more recent and advanced illustration is VX‐984, where deuterium incorporation effectively mitigates AO‐driven metabolism [9].

The development of d3‐l‐DOPA, represented by SD‐1077, has effectively demonstrated the impact on reactions catalyzed by MAO and dopamine β‐hydroxylases (DBH). Upon the decarboxylation of d3‐l‐DOPA by aromatic l‐amino acid decarboxylase, the metabolism of d3‐dopamine becomes modifiable through deuteration. Specifically, the incorporation of deuterium at the α‐carbon slows down MAO activity and the formation of d2‐3,4‐dihydroxyphenylacetic acid. Meanwhile, at the β‐carbon, it dampens DBH functionality, resulting in reduced levels of d2‐noradrenaline. This leads to the conclusion that d3‐l‐DOPA exhibits greater anti‐akinetic potency compared to l‐DOPA in a rodent model of Parkinson's disease without a concurrent increase in dyskinesias [78, 79, 80]. Considering that this modification is anticipated to influence dopamine metabolism at synapses, it aligns with expectations that the recently published Phase I clinical trial emphasized comparable peripheral pharmacokinetics and safety. This observation was made following a single oral dose of SD‐1077/carbidopa and l‐DOPA/carbidopa in healthy subjects, without any indication of a significant shift in the primary systemic metabolic pathways. Deuteration seems to influence the activity of other members within the amine oxidase family. This is illustrated by d2‐AVE5638, a tryptase inhibitor metabolized by semicarbazide‐sensitive amine oxidase, and d2‐lysine, serving as a substrate for lysyl oxidase [78, 81].

## 5 Opportunities for Deuterated Drugs

The therapeutic potential of deuterated drugs is enormous, as strategic H to D substitution can effectively enhance pharmacokinetic characteristics. These modifications often render increased metabolic stability, extended half‐life, and decreased toxicity, collectively increasing efficacy and patient compliance. The clinical pipeline indicates broad and growing applicability, incorporating candidates to treat cancer, neurodegenerative diseases, and viral infections. In tandem, the regulatory approvals and synthetic advances have enabled the use of deuterium at early stages of drug discovery. Driven by unmet clinical needs, an evolving regulatory landscape and robust industry relationship, deuterated drugs represent a promising prospect in the field of medicinal chemistry [82, 83].

### 5.1 Exploration of Therapeutic Areas Where Deuterated Drugs Show Promise

Deuteration of drugs is an approach that serves as a preventive measure against oxidative damage in cellular and vascular tissues, thereby mitigating the development of pathologies such as neurodegeneration, atherosclerosis, and macular degeneration. Ongoing research is elucidating the molecular mechanisms supporting these treatments, and there is potential for extending this platform to address nonoxidative diseases by incorporating deuterium‐reinforced amino acids and nucleobases [84]. Patients with COVID‐19 frequently encounter adverse effects from certain drugs stemming from both the parent compound and reactive metabolites. The exploration of deuterated versions such as (deupirfenidone or LYT‐100, deuterated oral remdesivir derivative VV116, molnupiravir, and many more) diverse medicinal compounds holds promise as potential therapeutics for COVID‐19, including isotopologues of repurposed or novel drugs [31, 85].

Lipid peroxidation significantly contributes to non‐neurological disorders, including atherosclerosis and retinal diseases. Deuterium‐reinforced linoleic acid (d‐PUFA) reduces lipid peroxidation and improves cognitive function in the Q140 mouse model of Huntington's disease. Treatment led to ∼80% lower striatal F2‐isoprostanes and better performance in memory tasks, although motor deficits and huntingtin aggregation were unchanged, highlighting its potential for targeting oxidative damage‐related cognitive impairment [86]. d‐PUFA trigger the phosphoinositide‐calcium signaling pathway in astrocytes, promoting their reactivation and exerting cytoprotective response on cortical neurons and astrocytes in vitro [87]. Additionally, d‐PUFAs effectively mitigated the development of atherosclerotic lesions in aortic valves in preclinical model of atherosclerosis. Additionally, d‐PUFAs may represent a viable target for addressing retinal diseases, such as age‐related macular degeneration (AMD) [88].

Deuterated docosahexaenoic acid (DHA), identified as a conditionally essential nutrient, exhibits a high susceptibility to peroxidation by reactive oxygen species. As such, it holds promise as a target for inhibiting DHA peroxidation and associated protein damage, potentially preventing the development of AMD [89]. Deuterium metabolic imaging (DMI) is a promising technique for noninvasive imaging of tumour metabolism, providing insight into tumour biology, treatment response, and prognosis assessment in a variety of metabolic pathways, potentially even in the context of immunotherapy. However, challenges such as quantification, safety, and hardware development need to be further investigated. DMI may be a more efficient imaging tool in clinical practice, and its advancement could contribute to precision medicine and to improving patient outcomes in the diagnosis and treatment evaluation of cancer [90].

Deuterium switches present numerous opportunities, notably in addressing challenges related to the pharmacological development of natural compounds, as exemplified by CYB003, a deuterated analogue of the natural product psilocybin. Although details about CYB003 remain undisclosed, it is currently undergoing testing in a Phase III trial for patients with major depressive disorder (NCT06564818) [91]. Similarly, multiple deuterated analogues of N, N′‐dimethyltryptamine (d8‐DMT), a serotonergic hallucinogen found in various plants, are in development to reduce MAO‐A‐mediated metabolism and attain inhalable formulations with increased stability for addressing psychiatric or neurological disorders [92].

### 5.2 Benefits Beyond Improved Pharmacokinetics: Efficacy, Safety, and New Opportunities

#### 5.2.1 Enhancing Therapeutic Index by Reducing Reactive Metabolites

Substitution of deuterium has the potential to significantly alter metabolic processes by the KIE when C–D bonds exhibit a slower rate of cleavage than the C–H bond. This decrease in metabolic turnover reduces the production of reactive intermediates that cause toxicity, such as electrophilic species or oxidative metabolites. In drugs that face the risk of being hepatotoxic due to metabolism or those that have an idiosyncratic adverse reaction, deuteration can be used as a molecular protective measure to stabilize potentially sensitive moieties without altering primary pharmacodynamics [6, 35]. This increased metabolic stability is reflected in an improved therapeutic index, allowing for increased efficacy at a lower risk. The strategy has shown its translational safety through such mitigation of reactive metabolite burden by antidepressants, antipsychotics, and kinase inhibitors as deuterated analogues [21, 34].

#### 5.2.2 Improving Target Selectivity and Reducing Off‐Target Effects

In addition to safety, the incorporation of deuterium can have an indirect positive effect on selectivity for molecular targets of interest by modulating binding kinetics in conjunction with receptor engagement. A deuterium label at a single substitution site can alter the lipophilicity, polar surface area, and hydrogen bonding profile of a compound, thereby directing the compound to efficient interactions at the active site of action and reducing interactions between the compound and an unwanted protein or enzyme [35, 93]. These changes can decrease negative pharmacological impacts, and cleaner signaling helps enhance efficacy and a valid dose response. From this perspective, deuteration is a fine‐tuning process that enables exact molecular recognition, a desirable characteristic in polypharmacological landscapes [6].

#### 5.2.3 Potential in Addressing Unmet Needs in Rare and Central Nervous System Disorders

The potential of deuterated compounds is significant in the treatment of rare and central nervous system diseases, where issues of drug exposure, blood–brain barrier penetration, and chronic dosage are substantial problems. Greater metabolic stability leads to increased central availability and a lower frequency of administration, thereby enhancing patient adherence in long‐term therapies. In addition, the reduced production of neurotoxic or reactive metabolites is particularly desirable in free‐standing neuropathology and conditions related to mitochondrial dysfunction. The predictable pharmacokinetic characteristics of deuterated drugs can therefore offer new treatment options under precision medicine, with reduced toxicity and long‐lasting treatment of conditions to which there are only a few therapies available [94, 95, 96].

### 5.3 Potential Applications in Rare and Neglected Diseases

Deuterated drugs are especially beneficial in the realm of rare and neglected diseases, where conventional drug development might face considerable obstacle. Deuterium‐enriched essential and conditionally essential biomolecules have been proposed for therapeutic applications to mitigate detrimental enzymatic reactions implicated in cancer and inflammatory diseases. Deuterated lysine, an essential nutrient, is a potentially safer option for anticancer interventions, given its dietary availability and integration into proteins through standard metabolic pathways [97]. In a separate investigation, deuterium‐enforced cytosine demonstrated a reduction in the rate of DNA methylation catalyzed by DNA methylases, pivotal in establishing and preserving DNA methylation patterns [98]. Aberrant DNA hypermethylation is closely associated with tumorigenesis, inflammation, and allergic responses [99]. Current interventions for these conditions involve DNA methylation‐modifying drugs such as azacytidine and its deoxy derivative decitabine. However, due to their inherent toxicity, there is a preference for less toxic cytosine derivatives with DNA demethylating capabilities as potential therapeutic agents, with deuterated cytosine being a candidate [98, 100]. Deuterated drugs can expedite clinical progress in diseases with limited research and development resources by building on nondeuterated drugs. However, introducing deuterated drugs in rare and neglected diseases requires thorough scrutiny of safety, efficacy, and feasibility. Addressing regulatory approval challenges and ensuring accessibility in resource‐limited settings is crucial for the effective incorporation of these therapies in these distinct disease contexts [27].

## 6 Analytical Methods for Deuterium‐Containing Compounds

The analytical methods for deuterium‐containing compounds are key tools in the development and discovery by leveraging advantages the physical and chemical properties of deuterium. Additional methods employed to characterize, quantify, and assess the metabolically profile of deuterated drugs include mass spectrometry and NMR spectroscopy [101, 102, 103]. Isotope labeling with deuterium is also utilized as internal standard for pharmacokinetic, metabolomic, and ADME studies and enhances analytical accuracy. More accurate determination of the incorporation and metabolic fate of isotopes can be achieved using advanced chromatographic techniques such as isotope ratio mass spectrometry. These tools help in the rational design of drugs with better pharmacokinetic and safety profiles when used clinically [103, 104].

Quantitative evaluation of deuterium holds significance across various scientific domains, including chemistry, biology, environmental science, and materials science. Deuterium, denoted as heavy hydrogen, is a hydrogen isotope distinguished by the presence of a neutron and a proton. A number of analytical methods are commonly used to detect deuterium and determine its content in chemical and pharmaceutical substances (Table 2). The popularity of mass spectrometry stems from its high sensitivity in detecting the mass difference between hydrogen and deuterium [105]. The use of NMR spectroscopy, especially, the 2H NMR, gives direct data regarding the presence and location of the deuterium atoms in a molecule [106]. It is also possible to use infrared spectroscopy since the C–D bonds have different frequencies than C–H bonds [107]. Separation and specific identification of deuterated compounds in complex mixtures are possible using coupled methods such as gas chromatography–mass spectrometry (GC–MS) and liquid chromatography–mass spectrometry (LC–MS) [108, 109]. Also, the high‐performance liquid chromatography (HPLC) is often utilized to separate and purify deuterated molecules [110]. In special research, neutron activation analysis (NAA) can also be applied in isotopic analysis and trace determination of deuterium in the samples [111].

**TABLE 2:** Analytical techniques for deuterium quantification.

Analytical technique	Principle/methodology	Applications	Advantage	Limitation	References
Mass spectrometry	Isotope ratio mass spectrometry (IRMS)	Organic compounds, water, materials	High‐precision measurement of isotope ratios	Expensive instrumentation; sample preparation may be required; limited to certain samp le types and size	[105]
Nuclear magnetic resonance (NMR)	Deuterium NMR	Structural and dynamic analysis of molecules	Nondestructive; provides structural information	Limited to certain types of samples; lower sensitivity compared with other methods	[106]
Infrared spectroscopy	Fourier transform infrared (FTIR) spectroscopy	Analysis of functional groups, compounds	Fast analysis; nondestructive	Sensitivity to sample matrix; may require calibration; less quantitative compared with mass spectrometry	[107]
Gas chromatography–mass spectrometry (GC–MS)	Isotope‐ratio GC–MS	Separation and analysis of compounds	High sensitivity; specific for labeled compounds	Sample volatility; potential for isotopic fractionation; requires calibration with standard materials	[108]
Liquid chromatography–mass spectrometry (LC–MS)	Isotope‐ratio LC–MS	Separation and analysis of compounds	Suitable for a wide range of compounds	Sample compatibility; potential for isotopic fractionation; requires calibration with standard materials	[109]
High‐performance liquid chromatography (HPLC)	Isotope‐ratio HPLC	Separation and analysis of compounds	High resolution; applicable to various samples	Limited to certain types of samples; requires careful optimization of HPLC conditions	[110]
Neutron activation analysis (NAA)	Prompt gamma activation analysis (PGAA)	Elemental analysis of deuterium‐containing samples	Nondestructive; high sensitivity	It requires access to neutron sources; there is potential for sample activation, but it is limited to specific types of samples.	[111]

Due to the unusual properties and uses of deuterium, the precise measurement of the deuterium content is of great importance in various scientific and industrial fields. In NMR spectroscopy, deuterium is an essential label to study molecular structure and dynamics, and the accurate measurement of deuterium content is critical to the interpretation of NMR data and thus analytical chemistry and biochemistry [112]. In chemical and biological research, deuterium‐labeled compounds are used as tracers to elucidate reaction mechanisms, metabolic pathways, and biological processes. In pharmaceuticals and drug development, deliberate deuterium incorporation in drug formulations can also change pharmacokinetics, which can lead to improved stability and efficacy [29]. In materials science and catalysis, precise measurement of the deuterium content is crucial for designing materials with specific properties. Safety and regulatory compliance in industries that use or generate deuterium is crucial for designing materials with specific properties and advancing science, technology, and knowledge of natural processes across various disciplines [6].

## 7 Biological and Toxicological Uncertainties: Metabolic Switching, Emergent Metabolite Profiles, and Associated Risks

Although the replacement of deuterium in drug molecules may increase metabolite stability and decrease toxic products, it also raises complex biological and toxicological questions that should be carefully explored in drug discovery and development paradigms. The altered physicochemical characteristics introduced by deuterium can transform the enzymatic landscape with previously unexpected switches in metabolism, resulting in new and distinctive toxicity profiles. This complexity is specifically acute with compounds that are highly metabolized through CYP450 enzymes, with varied networks of catalysis that have redundancy [65].

The likelihood of a biological uncertainty is primarily based on the KIE of deuterium, which decelerates the reaction site of a metabolic biotransformation. While this approach intends to control the formation of reactive intermediates at major metabolic “soft spots,” it can inadvertently redirect metabolism to unprotected regions, a phenomenon known as metabolic switching or shunting. This rerouting can yield a selective combination of metabolites, occasionally elevating some previously small or unusual outputs of biotransformation. These new metabolic pathways can generate metabolites with cases, some of which are incompletely characterized, such as in terms of their pharmacological or toxicological behavior, thereby increasing uncertainty. In compounds that have two or more pathways that may occur in the metabolic system, deuteration can have no effect on the total metabolism, instead of altering the activity among numerous possible enzymatic pathways, resulting in a ratio and profile of metabolites that are not predictable [35, 113, 114]. These uncertainties are further increased by any in vivo biological factors that include variable level of enzyme expression, organ distribution, and metabolic individual idiosyncrasy. Also, the deuteration of metabolites can alter metabolite polarity, stability, or tissue penetration, which may alter all changes in exposure in target and off‐target tissues—potentially increasing or decreasing the toxicity, or reducing efficacy. The complication needs a detailed risk evaluation that includes the characterization of the metabolites, biological activity profiling, and toxicological testing. Regulatory specifications with respect to deuterated drugs should, therefore, tackle these uncertainties using strong nonclinical testing and an appropriate safety study over a long‐term time.

To conclude, although deuterium integration is potentially beneficial in streamlining drug metabolism, it also provides a source of biological complexity and poses a safety hazard that requires a high level of scientific scrutiny. To obtain the maximum value of deuterium in modern pharmacotherapy, it is necessary to understand and pre‐empt the consequences of metabolic switches and the possible new toxicity. The deuterium replacement also enhances the safety and efficacy of drugs by improving metabolic stability, reducing the generation of toxic metabolites, and doubling the half‐life without altering the pharmacological properties. It allows logical design of drugs by reducing metabolic liabilities, optimizing drug regimens, and rediscovering drug candidates that had been limited or discontinued previously.

## 8 Challenges in Deuterium Drug Development

The development of deuterium drugs has mainly faced difficult synthetic issues regarding specific isotopic labeling (which may be technical and expensive). Complex methodologies are required to achieve high cell incorporation efficiency with minimal isotopic impurities. Moreover, there are unpredictable impacts on the metabolic pathways, such as metabolic switching and the appearance of new metabolites, which pose safety and regulatory challenges [35, 115]. Detailed toxicological testing is necessary to deal with new incidents of toxicity and off‐target effects. Clinical benefits over available treatments must be clearly demonstrated, unlike in research, which is a complex process due to the demands of regulatory agencies. On the whole, one can say that these scientific, technical, and regulatory challenges should be overcome in order to fully unlock the potential of deuterium‐based therapeutics [6].

Utilizing deuterium in drug discovery presents a compelling strategy with advantages and drawbacks. One significant benefit is its capacity to enhance a molecule's bond cleavage resistance while preserving steric hindrance and electronic properties. This sets it apart from other metabolic blockers, such as halides [116]. Additionally, deuterium may offer a safer alternative to fluorine as a bioisostere, which can give rise to species that pose potential risks to human health and the environment [117]. Deuteration, an intricate technique involving a rate‐limiting bond cleavage step, presents challenges in translating deuterated compounds from laboratory experimentation to practical medical applications. The failure of deuterated analogues in drug development can be attributed to various factors, including deuterium‐promoted multidirectional metabolic switching, diminished efficacy of metabolites in vivo, masking of deuterated K‐intercepted elimination by competing enzymes or nonmetabolic elimination mechanisms, and unpredictability in preclinical testing due to interspecies variability [118].

Despite the growing accessibility of deuterated reagents and synthetic methods, achieving high isotopic purity (preferably greater than 98%) in deuterated active pharmaceutical ingredients (APIs) remains a significant hurdle. Isotopic mixtures prove challenging to separate with conventional purification techniques [6, 101]. The synthesis of deuterated APIs at a scale sufficient for market supply is further complicated by the risk of under‐deuteration, which can be caused by suboptimal isotopic purity of reagents and inefficient labeling reactions. This may necessitate additional steps, repetition of the deuteration process, or deuterated solvents to prevent undesirable hydrogen‐to‐deuterium exchange [119].

The cost of deuterated reagents is higher than that of nondeuterated counterparts, and the availability of certain deuterated building blocks may be limited or involve specialized procedures, contributing to increased expenses and complexity in the synthesis process. Consequently, the manufacturing costs associated with deuterated APIs surpass those of nondeuterated counterparts, potentially rendering the synthesis of deuterated drugs financially challenging [6]. The absence of established guidelines from the US FDA or other regulatory bodies for addressing isotopic impurities in deuterated APIs raises concerns that regulatory agencies might default to using ICH Q3a and consider isotopologues and isotopomers as standard impurities [120]. This potential scenario could challenge companies to meet specifications for isotopic impurities, as these specifications rely on the isotopic purity of the deuterium sources or deuterated pools used in synthesizing APIs [121, 122].

## 9 Deuterium and the Pharmaceutical Industry

The contemporary landscape of deuterium within the pharmaceutical sector highlights a growing interest and investigation into compounds containing deuterium for drug development. Researchers and pharmaceutical entities are progressively utilizing deuterium, a stable hydrogen isotope, to manipulate the characteristics of drug molecules. Incorporating deuterium at specific positions within a drug's molecular structure can enhance metabolic stability, prolong half‐life, and influence pharmacokinetics, thereby potentially improving the effectiveness and safety profiles of pharmaceuticals [34]. Remarkably, specific deuterated drugs, such as deutetrabenazine (Austedo), have obtained regulatory approval or progressed to advanced clinical phases, illustrating the viability and potential of this strategy [123]. Companies are building portfolios of intellectual property including deuterated compounds and have patents for synthesis methods and molecular structures. While there are some potential benefits, challenges remain, including the complexity of synthesis and higher production costs. This landscape is evolving, with pharmaceutical companies of various sizes incorporating deuterium into their research pipelines and continuing to explore the potential applications of deuterium in drug design and development [35].

There is renewed interest in the field and several new companies developing and patenting deuterated versions of nondeuterated drugs are currently active (Deuterium Switch): Auspex (acquired by Teva in 2015 for $3.5B), Concert Pharmaceuticals, Deuteria Pharmaceuticals, and Deuterx. In addition, Retrotope is developing deuterated fatty acids resistant to lipid peroxidation, which may be used to alter the course of oxidative stress‐related diseases [124]. Deuterated drugs have seen significant progress since 2014, with the Teva acquisition of Auspex in 2015 for $3.5B, which brought a wide range of deuterated drug programs and intellectual property, including deutetrabenazine [23]. In fact, deutetrabenazine received US FDA approval in 2017 for the treatment of chorea in Huntington's disease, which represented the first regulatory approval of a deuterated drug [125]. The key clinical trial of deutetrabenazine showed significant symptom reduction over 12 weeks, which led to further exploration into uses for conditions such as Tourette syndrome and tardive dyskinesia [123]. The purchase of the rights to CTP‐656 by Vertex (a deuterated version of ivacaftor designed for use in cystic fibrosis) for an upfront payment of $160 million and up to $90 million in potential additional milestone payments highlights the growing interest and financial investment in deuterated drugs [126]. Several deuterium‐based drug candidates are presently undergoing clinical evaluation, as summarized in Table 3.

**TABLE 3:** Selected representative clinical trials involving deuterium‐based drug candidates.

Clinical trial number	Deuterated form	Parent drug	Status	Indication
NCT05262959	Donafenib	Sorafenib	Recruiting	Hepatocellular carcinoma, refractory differentiated thyroid Cancer, metastatic colorectal cancer
NCT01897896	Deutetrabenazine, (d_6_‐tetrabenazine; SD809; SD‐809, TEV‐50717)	Tetrabenazine	Completed	Chorea associated with Huntington disease
NCT02931838	BMS‐986165	Novel compound	Completed	Psoriasis
NCT02644278	VX‐984, 3 (M9831)	Novel compound	Completed	Advanced solid tumors
NCT02599792	CTP‐656, (d_9_‐ivacaftor, VX‐561, c‐10358, c‐10355)	Ivacaftor	Completed	—
NCT01458769	CTP‐518 (d_15_‐atazanavir)	Atazanavir	Completed	—
NCT02215499	JZP‐386 (d_4_‐sodium oxybate; C‐10323)	Sodium oxybate	Completed	—
NCT02960945	CTP‐543 (d_8_‐ruxolitinib)	Ruxolitinib	Completed	—
NCT02445794	RT001 (d_2_‐linoleic acid ethyl ester)	Linoleic acid	Completed	Friedreich's ataxia
NCT01328821	CTP‐499 (PCS‐499)	1‐[(S)‐5‐hydroxylhexyl]‐3,7‐dimethylxanthine (HDX)	Completed	—
NCT02229071	Donafenib (d_3_‐sorafenib; CM 4307)	Sorafenib	Completed	Advanced hepatocellular carcinoma
NCT05701995	Deucravacitinib (BMS‐986165)	—	Recruiting	Plaque psoriasis
NCT05478499	Deucravacitinib (BMS‐986165)	—	Active, not recruiting	Psoriasis
NCT04036435	Deucravacitinib (BMS‐986165)	—	Active, not recruiting	Psoriasis
NCT03924427	Deucravacitinib (BMS‐986165)	—	Completed	Psoriasis
NCT04167462	Deucravacitinib (BMS‐986165)	—	Completed	Psoriasis
NCT03599622	Deucravacitinib (BMS‐986165)	—	Terminated	Granulomatous colitis; Crohn's disease; Crohn's enteritis; granulomatous enteritis
NCT02763969	BMS‐986202	Pyridazine carboxamide derivative	Completed	Psoriasis
NCT05730725	BMS‐986322	—	Completed	Psoriasis
NCT05396105	Deucrictibant (PHA‐022121)	—	Recruiting	Hereditary angioedema
NCT02644278	VX‐984 (M9831)	—	Completed	Advanced solid tumors
NCT05124080	Deucravacitinib (BMS‐986165)	—	Not yet recruiting	Nail psoriasis

Data sources: ClinicalTrials.gov

Numerous collaborative initiatives and alliances in the development of deuterated drug demonstrate the diverse approaches used by academic organizations, pharmaceutical companies, and research institutions. The partnership between Concert Pharmaceuticals and Jazz Pharmaceuticals serves as an example. They collaborated to create CTP‐543, a deuterated form of ruxolitinib intended to treat alopecia areata. Through this strategic alliance, Concert Pharmaceuticals was able to take advantage of Jazz Pharmaceuticals’ expertise in dermatology and optimize the development of CTP‐543 95 [127].

## 10 Regulatory and Intellectual Property Issues

Regulatory and intellectual property systems of deuterated drugs have turned on US FDA structure‐based test of the so‐called active moiety, where a deuterated analog can be claimed as a new chemical entity granting a 5‐year NCE exclusivity (and, in specific cases, 7‐year orphan exclusivity) as it was granted with Austedo (deutetrabenazine). This case law permits the use of reference drug data under 505(b)(2) yet obtains the exclusivities, however, requires an evidentiary basis of non‐sameness or clinical superiority in the event of sameness is established. Patent strategy is narrower to particular deuteration patterns, reaction paths, and impurity profiles, but has to find a way to overcome obviousness and enablement issues as well as changing naming and impurity expectations by authorities (Table 4) [128]. The US FDA typically assesses drugs containing deuterium within the established regulatory framework applied to other pharmaceuticals. The regulatory procedure necessitates the submission of a New Drug Application or a Biologics License Application for biological products, with the choice depending on the inherent characteristics of the drug. The US FDA conducts a thorough review process encompassing safety, efficacy, and overall quality evaluations to ensure compliance with regulatory standards [129, 130, 131].

**TABLE 4:** List of representative patents for deuterium‐based compounds.

Patent number	Title	Patent holder	Technical field/target
EP2680843A2	Derivatives of pyrazole‐substituted amino‐heteroaryl compounds	Concert Pharmaceuticals Inc	Non‐small cell lung cancer (NSCLC) (Crizotinib)
US6221335B1	Method of using deuterated calcium channel blockers	Isotechnika Inc	Calcium channel blockers
US20090076025A1	Deuterium‐enriched dasatinib	Protia LLC	Tyrosine kinases inhibitor (dasatinib)
US20120264721A1	Analogues of cilostazol	Concert Pharmaceuticals Inc	Phosphodiesterase inhibitor
US20120141513A1	Deuterated fingolimod	Concert Pharmaceuticals Inc	Lysophospholipid edg1 (S1P1) receptor agonist
US20110257260A1	3,4‐methylenedioxyphenyl inhibitors of GABA aminotransferase and/or GABA reuptake transporter inhibitor	Auspex Pharmaceuticals Inc	Inhibitors of GABAaminotransferase and/or GABA reuptake transporter inhibitor
US20140018436A1	Deuterium‐enriched bupropion	Axsome Therapeutics Inc	Norepinephrine reuptake inhibitor and dopamine reuptake inhibitor
US20130245067A1	Deuterium‐enriched lenalidomide	Deuteria Pharmaceuticals Inc	Multiple myeloma
US20130090357A1	Deuterium‐enriched donepezil	DeuteRx LLC	Acetyl cholinesterase inhibitor
US20120302605A1	3‐Deutero‐pomalidomide	Deuteria Pharmaceuticals Inc	Multiple myeloma
US20120136037A1	Deuterium‐enriched ruboxistaurin	Protia LLC	Protein kinase C (PKC)‐b inhibitor
US20120122833A1	Deuterium‐enriched meropenem	Protia LLC	Antibiotic (bacterial infections)
US20110281952A1	Deuterium‐enriched tolterodine	Protia LLC	Antimuscarinic agent
US20110312983A1	Deuterium‐enriched alogliptin	Protia LLC	Selective dipeptidyl peptidase‐IV inhibitor
US20110281927A1	Deuterium‐enriched doripenem	Protia LLC	Injectable antibiotic
US20110201690A1	Deuterium‐enriched dapoxetine	Protia LLC	Selective serotonin reuptake inhibitor
US8524733B2	Benzoquinoline inhibitors of vesicular monoamine transporter 2	Auspex Pharmaceuticals Inc	Vesicular monoamine transporter 2 (VMAT2) inhibitors
US20160031801A1	Deuterated 2‐amino‐3‐hydroxypropanoic acid derivatives	Concert Pharmaceuticals Inc	NMDA glycine‐site antagonist
US20150210699A1	Deuterated ibrutinib	Sun Pharmaceutical Industries Inc	Bruton's tyrosine kinase (Btk) inhibitor
US10618884B2	Deuterated diaminopyrimidine compounds and pharmaceutical compositions comprising such compounds	Suzhou Zelgen Biopharmaceutical Co Ltd	Protein kinase‐associated diseases
ES2961843T3	Deuterated heterocycle‐condensed gamma‐carbolines as antagonists of 5‐HT2A receptors	Intra Cellular Therapies Inc	Antagonists of 5‐HT2A receptors
US5846514A	Enhancement of the efficacy of nifedipine by deuteration	Isotechnika Inc	Calcium channel blocker
US12291499B2	Deuterated tryptamine derivatives and methods of use	Cybin IRL Ltd	Disorder associated with serotonin 5‐HT2 receptor.
US10738036B2	Deuterated cystic fibrosis transmembrane conductance regulator (CFTR) modulators	Vertex Pharmaceuticals Europe Ltd	Cystic fibrosis

A key regulatory criterion for deuterated pharmaceuticals is unambiguous empirical evidence of clinical benefit. Although deuteration harnesses the KIE to prolong active half‐lives and extend metabolism, regulatory agencies require compelling evidence that these pharmacokinetic changes translate to tangible, real‐world benefits over the parent drug. Comprehensive pharmacokinetic and pharmacodynamic bridging studies are necessary to map the slowing of metabolic cleavage and ensure that a longer half‐life does not produce harmful plasma accumulation. Furthermore, extensive toxicity studies are required to monitor for metabolic switching, which occurs when the blockage of major clearance pathways drives the drug to metabolize via alternate, formerly unknown, or potentially harmful secondary pathways [6, 132]. The Chemistry, Manufacturing, and Controls (CMC) requirements for deuterated pharmaceuticals pose challenges, notably in terms of quality control standards for the deuteration site and deuterium isotope purity. Regulatory agencies mandate that sponsors provide a precisely specified isotopic distribution profile since it is extremely challenging to attain 100% isotopic purity during commercial‐scale synthesis. Pharmaceutical impurities are any nondeuterated or partially deuterated compounds that have the potential to drastically change the overall metabolic and toxicological profile of a drug. Thus, to ensure the precise location of deuteration, assess isotopic enrichment, and identify any unique degradation products, highly specialized analytical techniques like deuterium‐sensitive NMR spectroscopy and high‐resolution mass spectrometry are required [133].

## 11 Future Directions in Deuterium‐Containing Drugs

The outlook for deuterium in the pharmaceutical sector holds promise, with ongoing research and developments indicating potential impacts across various therapeutic domains. Foreseen trends encompass the diversification of drug portfolios as researchers investigate the advantages of incorporating deuterium in fields like oncology, neurology, and infectious diseases [134, 135]. The future of deuterium‐based drug discovery is poised to be a truly radical extension of human uses of perdeuteration, far beyond standard perdeuteration, which will be fueled by developments in precision chemistry, computer‐aided design, and a combination with other forms of therapy. Recent innovations in catalytic and late‐stage C–H activation chemistry have enabled the regio‐ and chemoselective deuteration of aromatic, heterocyclic, and aliphatic scaffolds, as well as the late‐stage functionalization of drug‐like cassettes without adverse effects on molecular size or functional group repertoire. These novel protocols (typically based on new iridium, silver or ruthenium catalysts, or customized organocatalytic motifs) offer increased control and extended substrate (scope) capabilities. Site‐selective deuteration, which allows for the selective protection of metabolically sensitive sites, overcomes the liability to the formation of toxic metabolites and obtains pharmacokinetic tuning much more effectively than universal isotope labeling. The strategy will become a giant in the new medicinal chemistry campaigns going forward and will surely grow as more explanation of the mechanism and innovation of catalysts is provided [30, 136, 137, 138, 139].

Artificial intelligence and machine learning are changing the logical design of deuterated molecules. AI platforms now have the capability to forecast metabolic hotspots, model the impact of isotope swaps on both binding and ADME profiles, and determine optimal patterns of deuteration to meet polypharmacology, toxicity, and synthesis constraints in silico through the use of sophisticated algorithms, deep learning, and quantum chemistry. New toolkit: Neighboring datasets of metabolism and deuteration of an inhaled drug can be used to prioritize target sites and predict the possible rerouting of the metabolite before experimental identification. Predictive modeling also facilitates dynamic feedback of medicinal chemistry and computational processes, leading to faster optimization of leads, reduced expenditures on experiments, and a data‐oriented and design‐make‐test‐analyze continuum. The combination of isotopic medicinal chemistry and AI is opening new avenues for the extremely efficient production of next‐generation, smart‐deuterated medicines that respond to even more stringent therapeutic and regulatory criteria [140, 141].

One of the most thrilling frontiers is the intersection with other advanced drug modalities, namely, proteolysis‐targeting chimaeras (PROTACs), peptide therapeutics, and oligonucleotide technology. Deuteration of metabolic soft spots in all four domains, both the linker and ligand domains, of PROTACs can increase metabolic stability, enhance systemic exposure, and reduce off‐target degradation, thereby enabling more efficient protein degradation activity [42]. These advantages have led to the increased use of site‐specific deuteration in peptide and peptidomimetic drugs, which can be targeted to prevent enzyme hydrolysis, improve oral bioavailability, and decrease rapid clearance, ultimately resulting in the creation of more effective peptide‐based therapies. Similarly, to enhance the utility of this isotopic technique, 7C deuterium can be incorporated into the backbone of oligonucleotides or into the sugar moieties to protect against nucleases or improve in vivo pharmacokinetics (WO2020160336A1). These techniques, which are currently at the preclinical and emerging clinical validation phases, demonstrate the synergistic value of deuterium in relation to the enhanced biopharmaceutical properties of distinct molecular systems.

With an overall increase in the fields of site‐selective deuteration, AI TM modeling, and modality convergence, the importance of deuterium chemistry appears to be undergoing a paradigm shift in drug design and optimization, no longer viewed as a niche modification to drug research methods, but as a pillar‐level field of pharmaceutical science. The combination of the deuterium toolkit allows medicinal chemists to make any rational override of metabolic restrictions, optimize drug properties, and expand therapeutic modalities hitherto hampered by pharmacokinetic constraints or toxicity liability issues. The ultimate role of deuterium, characterized by a deeper mechanistic insight and synthetic discovery developments, will be defined by its easy integration into discovery pipelines, international acceptability by regulatory bodies, and its future demonstration of clinical relevance to a wide range of human pathologies.

## 12 Conclusion

This review thoroughly explores the significance of deuterium in drug discovery, examining its historical roots and current applications, and discussing potential future developments. It explores the basic principles governing the integration of deuterium into drug structures, provides examples of successful cases, and elucidates the therapeutic possibilities it opens. Despite acknowledging challenges such as synthetic complexities and regulatory constraints, advancements in analytical methodologies and an increased understanding of deuterium's characteristics indicate promising prospects for this innovative methodology. Working together across industries is crucial to fully realize the benefits of deuterium‐based medicines. This collaboration will lead to more effective treatments and better patient outcomes. Going forward, the first area of research would involve clarifying the exact mechanistic pathways by which deuterium replacement affects multifaceted metabolic pathways and delays the onset of clinical effects. Further application of deuterium chemistry, by incorporating its use into current technologies such as artificial intelligence‐based drug design, biomarker‐based precision therapies, and sophisticated isotopic analytical platforms, will continue to accelerate its translation potential. Finally, long‐term interdisciplinary partnerships and increased clinical validation are required to make deuterium‐enabled therapeutics a cornerstone of next‐generation drug development and personalized medicine.

## Author Contributions

This literature survey and data collection were done by Mukta Lele and Ajit Manchare, who were equally involved with drafting the first manuscript. Swapnali Parit contributed to figure preparation. Amol Gholap helped arrange the pharmaceutical and formulation‐related materials and critically revised the relevant sections. Krishna Jadhav contributed by critically editing and revision of the manuscript. Satish Rojekar helped conceptualize the review, interpret its pharmacological features, and provide an intellectual review of the manuscript. Navnath Hatvate edited the work, helped organize the manuscript, interpreted the data, and edited the final version. Keshav Raj Paudel contributed to concept development, critical scientific review, and manuscript refinement. All authors have read and approved the final version of manuscript.

## Funding Information

The Authors have nothing to report.

## Ethics Statement

The authors have nothing to report.

## Conflicts of Interest

The authors declare no conflicts of interest.

## Data Availability

The authors have nothing to report.
